# Establishing quantitative real-time quaking-induced conversion (qRT-QuIC) for highly sensitive detection and quantification of PrP^Sc^ in prion-infected tissues

**DOI:** 10.1186/2051-5960-1-44

**Published:** 2013-08-02

**Authors:** Song Shi, Gerda Mitteregger-Kretzschmar, Armin Giese, Hans A Kretzschmar

**Affiliations:** 1 Center for Neuropathology and Prion research, Ludwig-Maximilians-University, Munich, Germany

**Keywords:** Prion, PrP^Sc^, PrP^27-30^, Quantitative RT-QuIC

## Abstract

**Background:**

PrP^Sc^, the only known constituent of prions, the infectious agents causing prion diseases, can be detected by real-time quaking-induced conversion (RT-QuIC). However, there is no efficient method to quantify the amount of PrP^Sc^ by RT-QuIC.

**Results:**

Here we introduce quantitative RT-QuIC (qRT-QuIC) to quantify with high accuracy minute amounts of PrP^Sc^ in the brain and various peripheral tissues at levels far below detection by *in vivo* transmission. PrP^Sc^ is relatively resistant to treatment with proteinase K (PK). However, as there can also be a fraction of pathological PrP that is digested by PK, we use the term PrP^27-30^ to denote to the amount of PrP^Sc^ that can be detected by immunoblot after PK treatment. qRT-QuIC is based upon the quantitative correlation between the seeded amount of PrP^27-30^ and the lag time to the start of the conversion reaction detected by RT-QuIC. By seeding known amounts of PrP^27-30^ quantified by immunoblot into qRT-QuIC a standard calibration curve can be obtained. Based on this calibration curve, seeded undetermined amounts of PrP^27-30^ can be directly calculated. qRT-QuIC allowed to quantify PrP^27-30^ concentrations at extremely low levels as low as 10^-15.5^ g PrP^27-30^, which corresponds to 0.001 LD_50_ units obtained by *in vivo* i.c. transmission studies. We find that PrP^27-30^ concentration increases steadily in the brain after inoculation and can be detected at various time points during the incubation period in peripheral organs (spleen, heart, muscle, liver, kidney) in two experimental scrapie strains (RML, ME7) in the mouse.

**Conclusions:**

We suggest that an automatic quantitative system to measure disease progression as well as prion contamination of organs, blood and food product is feasible. Moreover, the concept of qRT-QuIC should be applicable to measure other disease-associated proteins rich in β-pleated structures (amyloid) that bind ThT and that show seeded aggregation.

## Background

Prion diseases are a group of transmissible neurodegenerative lethal disorders in humans, cattle, sheep, elk, mink and experimental animals. These diseases are characterized by neuronal death and the accumulation of pathological disease-associated prion protein (PrP^Sc^) in the central nervous system [1]. PrP^Sc^ is thought to be the essential, if not the exclusive, component of the transmissible agent, or prion. Prion propagation seems to rely on autocatalytic amplification of PrP^Sc^ by converting the host-encoded cellular prion protein (PrP^C^) to the pathogenic PrP^Sc^ form without the participation of nucleic acids [2,3]. The conversion of PrP^C^ to PrP^Sc^ is a post-translational event and involves a conformational change of the protein [4,5]. To distinguish between PrP^Sc^ which is isolated from infectious tissue and is *per definitionem* associated with the TSE agent on the one hand and structurally altered PrP, which has been converted into a ProteinaseK-resistant form *in vitro*, on the other, we refer to the latter as ‘PrPres’. The most commonly used approach to distinguish PrP^Sc^ from PrP^C^ when analyzing infected tissues is based on pre-treatment with proteinase K as PrP^Sc^ is relatively PK-resistant. However, as there can also be a fraction of pathological PrP that is digested by PK treatment [6], we use the term PrP^27-30^[2] in this manuscript whenever we refer to the amount of PK-resistant PrP^Sc^ that can be detected by immunoblotting.

**Figure 1:**
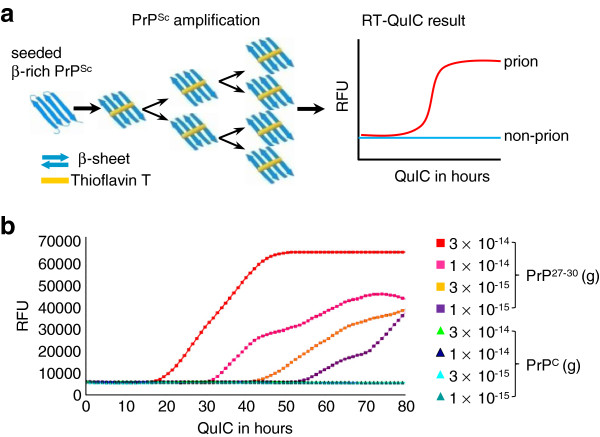
**The basis of amplifying PrP**^**Sc **^**with RT-QuIC. (a)** Schematic illustration of RT-QuIC. PrP^Sc^ converts rPrPsen to rPrPres thereby increasing the total amount of β-sheeted PrP. This increase can be demonstrated by the increased value of ThT-fluorescence. Therefore, the sample containing PrP^Sc^ (prion) is distinguished from that without PrP^Sc^ (non-prion). RFU, relative fluorescence units. **(b)** However, it was not the amount of newly formed rPrPres but the time to reach the steep increase of amplification (ascendant curves) that was related to the seeded quantity of PK-treated PrP^Sc^ (PrP^27-30^). Different amounts of purified mouse RML scrapie-prion PrP^27-30^ and normal mouse PrP^C^ were seeded into reactions to perform 80 hours of RT-QuIC. PrP^C^ did not cause ascendant curves.

The infectivity of prions presents a serious risk to human health. One important issue in prion research is the sensitive detection and quantification of prions at low levels during the incubation period and in peripheral tissues to provide suitable detection assays for medicine and bio safety. Real-time quaking-induced conversion (RT-QuIC) was established to detect prion infectivity with very high sensitivity from diluted brain [7], from cerebrospinal fluid (CSF) of end-stage disease containing only very low levels of infectivity [8-10], and from CSF during the incubation period [11]. The mechanism of RT-QuIC is based upon the conversion of PK-sensitive recombinant prion protein (rPrPsen) into a PK-resistant rPrP (rPrPres) conformer, which is rich in β-sheet structures, by seeding the reaction mixture with PrP^Sc^ and periodic shaking (Figure 1a). The correlation between Thioflavin T (ThT) fluorescence and rPrPres has been shown previously and is used to monitor the conversion in a real-time curve [8].

Although protease-resistant PrP^27-30^ is often used as a definitive biological marker for TSE infection, the titer of infectivity measured by inoculation in experimental animals does not always fully correlate with the amount of PrP^27-30^ detected by immunoblot. The correlation of the seeding activity on rPrPsen conversion into rPrPres measured by RT-QuIC with the amount of PrP^27-30^ is also a complex issue. For example, it has been shown that vCJD prions have less seeding activity than sCJD prions despite the relatively high PrP^27-30^ concentration [10], and that prions from brains of 263 K-affected mice with little immunoblot-detectable PrP^27-30^ have a seeding activity comparable to that associated with the high-PrP^Sc^ strain, 139A [12]. In previous studies, we showed that the complex correlation between amounts of PrP^27-30^ and infectivity *in vivo* and seeding activity in vitro can be explained at least in part by differences in size distribution of PrP aggregates [13]. Consequently, RT-QuIC seeding activity may correlate more closely with prion infectivity than with PrP^27-30^ levels, which can be considered an advantage in regard to the development of assays for prion detection.

We observed that in the RT-QuIC reaction, adding small amounts of PrP^Sc^ resulted in a delayed initiation of conversion detected by ThT fluorescence (Figure 1b). This delay and the shape of the resulting ThT fluorescence curve appeared similar to the kinetics of amplification of DNA that is used for quantitative RT-PCR analysis [14,15]. Thus we investigated whether real-time protein amplification was quantitative and established a highly sensitive assay for the quantification of prion infectivity in a high-throughput system based on measuring lag time of detectable conversion. We termed this approach qRT-QuIC and show that it allows quantitation of prions in various tissues with a detection limit corresponding to 0.001 LD_50_ units.

## Results and discussion

### Establishing quantitative RT-QuIC

**Figure 2:**
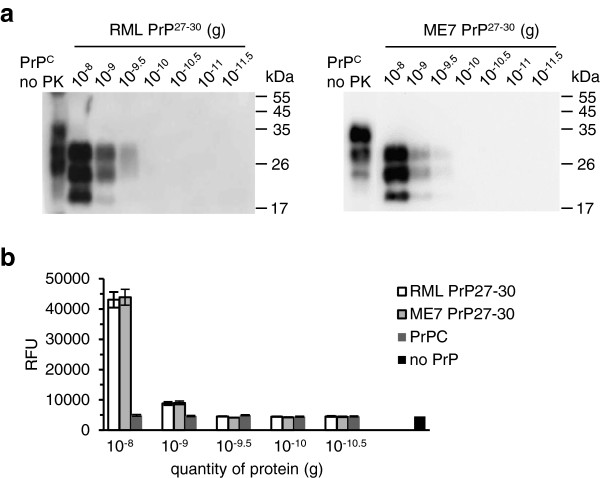
**Directly detecting purified PrP**^**27-30 **^**with immunoblotting and ThT-binding fluorescence. (a)** Purified mouse RML and ME7 scrapie-prion PrP^27-30^ was serially diluted and detected by immunoblotting. Aliquots were digested with 100 μg/ml Proteinase K (PK) before loading on the gel. Undigested PrP^C^ is shown as a migration control. Signals were detected by 4H11 monoclonal antibody. M_r_ is shown on the right. **(b)** Fluorescence of purified RML and ME7 PrP^27-30^ and normal PrP^C^ was measured after ThT-binding. The mean and s.e.m. are shown (n = 5).

To establish a high-throughput quantification system, PrP^27-30^ derived from two mouse-adapted scrapie prion strains, RML and ME7, was purified from infected C57BL/6 mouse brain by repeated NaCl precipitation allowing recovery of 97% of the total PrP^Sc^[16]. The concentration of protease-resistant PrP^Sc^ was estimated by semi-quantitative immunoblotting and comparing band intensities to reference samples containing known quantities of rPrPsen [17] (data not shown). To estimate the minimum amount of PrP^27-30^ equivalent that can start the seeding reaction, we serially diluted PrP^27-30^ (from 10^-8^ to 10^-11.5^ g) (Figure 2a). 10^-9.5^ g of both RML and ME7 PrP^27-30^ were visible as a faint band on the immunoblot while 10^-10^ g was not detectable. As the control, PrP^C^ from healthy C57BL/6 mouse brain was purified [18] and quantified as above. For controlling the quality of the real-time curve, the ThT-binding fluorescence of both PrP^27-30^ and PrP^C^ was measured (Figure 2b); the results showed that the fluorescence starting from 10^-9.5^ g of PrP^27-30^ was identical to that of PrP^C^ and the blank (no PrP). Since 10^-10^ g of PrP^27-30^ from both prion strains was undetectable in either immunoblot or ThT-fluorescence, we chose it as the initial seed.

**Figure 3:**
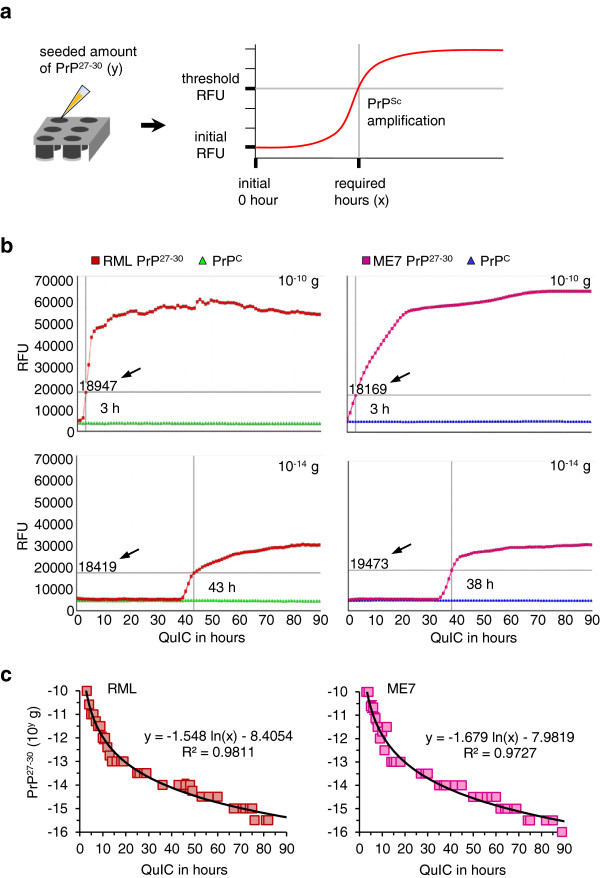
**Establishing the quantitative RT-QuIC (qRT-QuIC). (a)** Schematic illustration of qRT-QuIC. The PrP^27-30^ propagating duration (hour) required to reach the threshold which was at least 3 times the starting fluorescence was set as the independent variable (x), the correlated seeded amount of PrP^27-30^ was the dependent variable (y). **(b)** Different amounts of purified PrP^27-30^ (10^-10^ to 10^-16^ g with serial 10^0.5^-fold dilution) were seeded into RT-QuIC reactions using mouse rPrPsen as the substrate. The RT-QuIC process was followed from 0 to 90 h by showing the number of hours required to reach the threshold (indicated by black arrows and intersecting lines). Purified PrP^C^ with identical amounts was seeded in independent RT-QuIC reactions as control. Seeded amounts of both PrP^27-30^ and PrP^C^ are indicated on the top right; see also Additional file 1: Figures S2 and S3. **(c)** The results from five repeats of RT-QuIC seeded by PrP^27-30^ were provided to yield standard calibration curves and formulas for quantification. This relates QuIC time necessary to reach the threshold to the amount of seeded PrP^27-30^. The mean and s.e.m. are shown (n = 5).

To establish the quantitative RT-QuIC (qRT-QuIC), the seeds containing 10^-10^ to 10^-16^ g of purified RML and ME7 PrP^27-30^ and PrP^C^ per 10 μl were prepared by serial half-log (10^0.5^-fold) dilution (a total of 39 samples). Full-length mouse rPrPsen (amino acids 23–230) was utilized as the substrate. To estimate the time-span of the reaction, 130 h of RT-QuIC was tested for determining the appropriate duration. Spontaneous conversion seemed to occur after 90 h (Additional file 1: Figure S1). For standardizing the results, a ‘positive reaction’ of RT-QuIC was recorded when the detected intensity of fluorescence was equal or higher than a threshold. The threshold was 3 times of the fluorescence of the initial phase (0 h). The corresponding time (hours) required to reach the threshold was recorded as the independent variable (x), and the equivalent amount of seeded PrP^27-30^ was the dependent variable (y), as shown in Figure 3a. Next, RT-QuIC reactions seeded with 10^-10^ to 10^-16^ g of PrP^27-30^ and PrP^C^ were performed up to 90 h (shown in Figure 3b and Additional file 1: Figures S2 and S3). Five repeats of RT-QuIC for each seeded amount of PrP^27-30^ and PrP^C^ equivalents (a total of 260 reactions) were tested. Reactions seeded with 10^-10^ to 10^-15.5^ g of PrP^27-30^ were positive within 90 h whereas most of those seeded with 10^-16^ g PrP^27-30^ were negative (Additional file 1: Figure S4). Reactions seeded with different amounts of PrP^C^ did not show spontaneous conversion up to 90 h. Therefore, the RT-QuIC allowed detecting 10^-15.5^ g (≈ 0.32 fg) of PrP^27-30^, which was 1 million times more sensitive than the immunoblot shown in Figure 1a. By analyzing the distribution of the required hours mathematically using a standard tool (Microsoft Excel), we obtained calibration curves and derived two formulas for calculating the quantities of seeded RML and ME7 PrP^27-30^ in the qRT-QuIC system (Figure 3c).

### Measuring PrP^27-30^ concentrations in brains and peripheral organs at different dpi of prion infection

**Figure 4:**
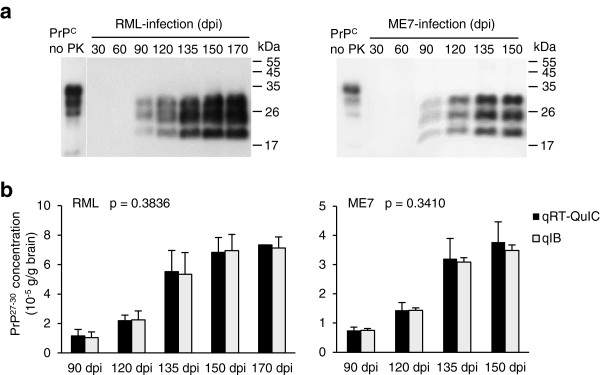
**Quantification of RML and ME7 PrP**^**27-30 **^**in brain at various days post infection (dpi). (a)** 1 mg of brain from scrapie-infected mice at was analyzed by immunoblotting after digestion with 100 μg/ml PK. PrP^C^ is shown as a migration control. Signals were detected by 4H11 monoclonal antibody. M_r_ is shown on the right. **(b)** Comparing PrP^27-30^ concentrations measured by quantitative RT-QuIC and quantitative immunoblot. The brains of RML-inoculated mice 90 dpi to 170 dpi and ME7-inoculated mice 90 to 150 dpi were chosen regarding the capacity of immunobloting to detect PrP^27-30^ from the time-point not earlier than 90 dpi. The significance (p value) was calculated with two-way ANOVA by using detecting methods and time-points as the variables. Shown data are mean and s.e.m (n = 5).

To see the feasibility of qRT-QuIC to determine the progression of prion disease, qRT-QuIC was used to measure PrP^27-30^ at different days post inoculation (dpi). 30, 60, 90, 120, 135, 150 and 170 dpi were chosen for RML, 30 to 150 dpi were chosen for ME7, since 170 dpi denotes the terminal stage of the RML strain while 150 dpi is the terminal stage of the ME7 strain. Groups of each 5 C57BL/6 mice were inoculated intracerebrally and the brains and peripheral tissues (heart, liver, spleen, lung, kidney and hind-limb muscle) were harvested at the dpi indicated above. The tissues of age-related healthy C57BL/6 mice were chosen as controls (n = 5 for each dpi). 10 mg of each tissue were treated with the method of purifying PrP^27-30^ for obtaining tissue extracts as the seeds of RT-QuIC reactions. This purification step was important to remove potential components affecting RT-QuIC efficiency. Since the PrP^27-30^ level is much higher in the brain than in other organs, 1 mg of brain from infected mice of different time-points was first analyzed by immunoblotting for obtaining an overview of the presence of PrP^27-30^ (Figure 4a). By immunoblotting, neither in RML nor in ME7 was PrP^27-30^ detectable at 30 and 60 dpi, in both strains PrP^27-30^ first appeared at 90 dpi and showed a steady increase to the terminal stage. For the preparation of RT-QuIC seeds, the brain extracts of 90 to 170 dpi of the brain were diluted 10^4^-fold, and those of the spleen and muscle were diluted 10-fold. Before measuring PrP^27-30^ concentrations in peripheral organs, a comparison was done to estimate the reliability of qRT-QuIC (Figure 4b). The results showed that PrP^27-30^ concentrations in the brains obtained from qRT-QuIC were comparable to those from quantitative immunoblottings at different time points after inoculation (see also in Additional file 1: Table S1), indicating that qRT-QuIC is suited to measure the PrP^27-30^ concentrations.

**Figure 5:**
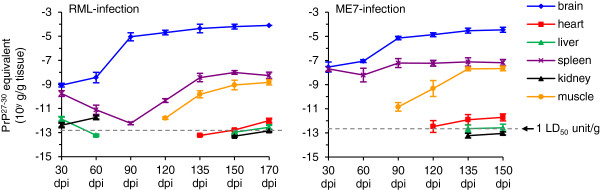
**The seeding activity (expressed as PrP**^**27-30 **^**equvalents in g/g tissue) in various tissues from different time-points after inioculation were measured by qRT-QuIC by using the formulas shown in Figure**3**c.** The PrP^27-30^ concentration corresponding to 1 LD_50_ units per gram of tissue is represented by a gray broken line. Shown are the mean and s.e.m. (n = 5).

After 90 h of amplification we calculated the concentration of PrP^27-30^ equivalents based on the detected seeding activity by using the formulas in Figure 3c. The levels of PrP^27-30^ equivalents (i.e. seeding activity) in the brain showed the expected increasing tendency (Figure 5). In particular, seeding activity reached the highest levels at 170 dpi in the RML strain and 150 dpi in the ME7 strain. The detection of seeding activity from 30 to 90 dpi of both prion strains was negative in the heart and hind-limb, but the signals from the heart started to show at 135 dpi in the RML strain and 120 dpi in the ME7 strain, while those from muscle were positive starting at 120 dpi of the RML strain and 90 dpi of ME7. Interestingly, seeding activity in the liver and kidney in the RML strain was detectable in early stages (30 and 60 dpi), and disappeared during the intermediate stages of infection (90, 120 and 135 dpi), whereas those of ME7-infection were continuously negative till 135 dpi. Seeding activity in the spleen of both prion strains was decreasing at early stages followed by an increase in the intermediate stages. The concentration of PrP^27-30^ equivalents in all three tissues was increased at late to final stages. No seeding activity was detected in the lungs at any time. Since qRT-QuIC was 1 million times more sensitive than normal immunoblotting, these results suggest a possibility of using qRT-QuIC to track disease progression or analyze prion propagation in various tissues.

To demonstrate the feasibility of qRT-QuIC in the assessment of bio-hazard risks, the concentration of PrP^27-30^/g tissue (g/g) in various tissues measured by qRT-QuIC was compared to the reported half-lethal doses (LD_50_) of both prion strains. The known LD_50_ of RML and ME7 are 10^-8.8^ and 10^-8.3^ g of terminal brains, respectively [19]. Using the PrP27-30 concentration measured by qRT-QuIC, we can roughly extrapolate that 1 LD_50_ unit contained 10^-12.93^ g of PrP^27-30^ for RML and 10^-12.72^ g for ME7. One LD_50_ unit is shown as the gray dotted lines in Figure 5 to indicate the extent of prion concentration in each gram of tissue (see also Additional file 1: Tables S2 and S3), suggesting that qRT-QuIC can be used for estimating prion contamination in biological materials. As our protocol uses an extraction method for PrP^27-30^ prior to qRT-QuIC that removes potentially interfering factors present in peripheral tissues, the calibration curve obtained for brain-derived PrP^27-30^ should also provide meaningful results for other tissues from the same species. Moreover, our findings for RML and ME7 indicate that similar assay conditions can be efficient for different strains. However, to ensure optimum sensitivity, the exact conditions of the qRT-QuIC assay need to be established for different species and strains.

In a recent study, Wilham and colleagues [7] used SD50 (50% seeding dose) to quantify seeding activity in the RT-QuIC. SD50 was defined as minimum seeded amount causing 50% of RT-QuIC reactions to be positive. Using end-point titration in a dilution series for quantification, SD50 was shown to correlate with the infectivity of 263 K strains in hamster. To obtain SD50 values for one prion strain in RT-QuIC, serially diluted standard sample are required, e.g., serially diluted prion-infected reference brain homogenate and serially diluted undetermined materials. Thus, this approach requires multiple repeats to yield the percentage of positive reactions for every dilution. In contrast, qRT-QuIC is a lag time-based assay and the amount of PrP^27-30^ equivalents in suspected materials can be directly calculated and quantified with much fewer repeats based on the calibration curve. The respective advantages and disadvantages of end-point titration and lag time assays are well known from assays of prion infection *in vivo*. End-point titration provides an accurate measure of infectious units. However, end-point *in vivo* assay also requires multiple repeats for each dilution of seeds to obtain the titration of one prion strain, whereas the incubation time *in vivo* assay is more commonly used for prion quantification as much less animals are required. Hence, we conclude that the qRT-QuIC assay which uses lag time is more suitable than SD50 measurements for quantitative detection and high-throughput assay for prion diagnosis.

## Conclusion

In conclusion, qRT-QuIC is a new advancement of the RT-QuIC system, which enables us to quantify PrP^27-30^ concentrations at extremely low levels as low as 10^-15.5^ g PrP^27-30^, which corresponds to 0.001 LD_50_ units. qRT-QuIC is based upon the quantitative correlation between the seeded amount of PrP^27-30^ and the lag time to the start of the conversion reaction detected by RT-QuIC. The principle of generating standard curves by monitoring the fluorescence and setting threshold levels makes qRT-QuIC applicable to the development of an automatic system similar to quantitative RT-PCR. qRT-QuIC can be used as a rapid and reliable novel research tool, increase the precision of diagnosis, and help to follow the effects of therapy of prion diseases. Moreover, this concept should be applicable to measure other proteins rich in β-pleated structures (amyloids) that bind ThT and that show seeded aggregation.

## Methods

### rPrPsen expression and purification

BL21 (DE3) E.coli and pET41a system (Merk, Germany) were used to express mouse rPrPsen. Bacteria were cultured in LB medium at 37°C with 220 rpm shaking and were added 1 mM of IPTG (final concentration) when OD reached 0.7 to 0.9. The cell pellet was harvested by 10,000 g centrifugation at 4°C for 10 min after 4 hours induction. Thereafter, cells were resuspended by BugBuster Master Mix (Novagen, Germany) containing rLysozyme and Benzonase for extracting inclusion bodies (manual for BugBuster, Novagen, Germany). The extracted inclusion bodies were denatured with 8 M Guanidine hydrochloride at 25°C for 1 hour. The denatured protein solution was centrifuged at 16,000 g at 4°C for 20 min to remove the debris.

We chose Ni^2+^-NTA superflow (Qiagen, Germany) resin for protein purification. The NTA resin was prepared by following the manufacturer’s manual. The denatured protein was loaded onto prepared resin followed by binding with inversion on the rotor at 25°C for 1 hour. After that, the resin was loaded into the column (GE healthcare, USA) followed by connecting to the AKTA prime (GE healthcare, USA). The followed purification procedures including refolding and elution were performed as described [7].

After elution, the rPrPsen solution was loaded into 6 KDa Cellu Sep dialysis tubing (Interchim, France) followed by immersion in pre-chilled dialysis buffer (9 mM NaH_2_PO_4_, 1 mM Na_2_HPO_4_, pH 5.9) at 4°C. The dialysis was done in 2 successive steps, which were 2 hours and 18 hours, respectively. 100 volumes of dialysis buffer were used for each step. The dialyzed solutions were sterilized with a 0.22 μm filter (Millipore, USA) and the absorption was measured at 280 nm for calculating the concentration of rPrPsen. The concentration of each fraction was adjusted to 0.5 mg/ml by adding sterilized and chilled dialysis buffer. The rPrPsen solution was aliquoted and frozen in liquid nitrogen, followed by transfer to a freezer (-80°C) for long-term storage.

### Tissue preparation

6-week old C57BL/6 mice were inoculated with mouse-adapted RML and ME7 scrapie (i.c). 10 μl of 10% brain homogenate in 1 × PBS (pH 7.2) was used for each inoculum. 7 time-points (30, 60, 90, 120, 135, 150 and 170 dpi, days post inoculation) were set for RML-infection and 6 time-points (30, 60, 90, 120, 135 and 150 dpi) were for ME7-infection. At each dpi, five mice were sacrificed with CO_2_. To harvest the tissues (brain, heart, liver, spleen, lung, kidney and hindlimb muscle) they were washed with chilled 1 × PBS containing 5% sodium citrate to remove the blood. Then tissues were weighed and stored in liquid nitrogen. The tissues of age-related control C57BL/6 mice were prepared following the same procedure.

### PrP^27-30^ purification

We followed a published protocol [16] to purify PrP^27-30^. The RML- and ME7-infected mouse tissue was prepared to 10% homogenate (w/v) with lysis buffer (pH 7.2) containing 130 mM NaCl, 10 mM NaH_2_PO_4_, 10 mM Na_2_HPO_4_, 0.5% Triton X-100, 0.5% sodium deoxycholate, 2 mM MgCl_2_, 2.5 U/ml of Benzonase (Merck, Germany) and EDTA-free protease inhibitor cocktail (Roche, Switzerland). Homogenate was incubated at 25°C for 30 min for digesting nucleic acids followed by 1,000 g centrifugation at 4°C for 5 min for removing debris. Thereafter, 100 μl of supernatant were doubly diluted with lysis buffer to reach 200 μl of total volume followed by 20 μg/ml of PK-digestion at 37°C for 1 h. The digestion was stopped by adding 5 mM PMSF (Sigma-Aldrich, Switzerland) and then the PK-treated supernatants were transferred into 300 μl of 1 × QuIC buffer (130 mM NaCl, 5 mM NaH_2_PO_4_, 5 mM Na_2_HPO_4_ and 1 mM EDTA, pH 7.4). The total volume reached 500 μl.

The preparations were then brought to equal volume of buffer containing 20% NaCl and 0.1% sarkosyl. These solutions were vortexed vigorously followed by incubating on ice with gentle shaking for 10 min. After centrifugation at 16,000 g at 4°C for 10 min, the pellets were washed by 500 μl of 20 mM Tris–HCl containing 0.05% sarkosyl followed by 16,000 g of centrifugation at 4°C for 10 min. This washing step was repeated twice. The pellet was stored at -80°C till being used as the seed.

For preparing the prion seeds, the frozen pellets were thawed at 4°C followed by washing with 500 μl of ddH_2_O. The resuspended solutions were precipitated by centrifugation at16,000 g for 10 min at 4°C. This step was repeated twice. The last pellets were resxuspended thoroughly by 50 μl of ddH_2_O followed by 1,000 g of centrifugation at 4°C for 1 min. 45 μl of supernatant from peripheral tissues (tissue extract) was brought to qRT-QuIC to be both the seed and required water. 10 μl of PrP^27-30^ with known concentration quantified by semi-quantitative immunoblotting [17] was used as the seed for obtaining standard a calibration curve.

### PrP^C^ purification

We referred to a published protocol [18] to purify mouse PrP^C^. The brain from a healthy 20-week old C57BL/6 mouse was homogenized in chilled 1 × PBS (pH 7.2) containing EDTA-free protease inhibitor cocktail (Roche, Switzerland) to make a 10% homogenate (w/v). After centrifugation at 3,000 g at 4°C for 30 min, the pellet was resuspended with an equal volume of chilled buffer (pH 7.2) containing 130 mM NaCl, 10 mM NaH_2_PO_4_, 10 mM Na_2_HPO_4_, 2% NP-40, 1% sodium deoxycholate and EDTA-free protease inhibitor cocktail (Roche, Switzerland). After incubation on ice for 30 min, the homogenate was subjected to 100,000 g centrifugation at 4°C for 30 min to remove the debris. The supernatant was filtrated with a 0.22 μm filter (Millipore, USA) followed by pouring over the ImmunoPure Immobilized Protein A column (Pierce, USA) to remove the endogenous immunoglobulins. The filtrated solution was incubated with 1 ml of Protein A resin cross-link to mouse-PrP specific monoclonal 4H11 antibody at 4°C over night. Then the resin was washed with 10 volumes of washing buffer (20 mM Tris–HCl, 500 mM NaCl and 5 mM EDTA, pH 8.0) followed by washing with 10 volumes of 1 × PBS containing 0.5% NP-40. PrP^C^ was eluted with 5 ml of 200 mM glycine. The solution was loaded into 10 kDa filter centrifugal tubes (Millipore, USA) to centrifuge at 3,000 g at 4°C for 1 h for desalting and buffer exchanging. Then the protein was resuspended with 5 ml of 1 × QuIC buffer and subjected to gel filtration by passing over the Superdex 75 column (GE lifescience, USA). The harvested protein in the peak was concentrated with a 10 kDa filter centrifugal tube (Millipore, USA) followed by estimating protein concentration with quantitative immunoblot. The yield of mouse PrP^C^ was approx. 3 μg/brain.

### Quantitative RT-QuIC

Preparations for quantitative RT-QuIC were loaded into a Nunc 96-well plate (Thermo Scientific, USA) in a bio-safety cabinet. The plate was sealed with transparent tape for avoiding cross-contamination and aerosol. Reactions were performed on a FLUOstar Optima (BMG Labtech, Germany) at 37°C for 90 hours with 1 min shaking at 600 rpm followed by 1 min stationary incubation. The fluorescence was measured with bottom optic every hour. Excitation was 440 nm, emission was 480 nm and the gain setting was 2000. Curves and intersecting lines (for indicating both the threshold and required hour) were directly shown by the provided functions of BMG Optima Data Analysis software. Other details are shown in the following Method Table 1.

**Table 1:** The ingredients of qRT-QuIC preparation

**Component**	**volume**
10 x QuIC buffer	10 μl
(100 mM NaH_2_PO_4_, 100 mM Na_2_HPO_4_, 10 mM EDTA, 1.3 M NaCl, pH 6.9)	
dd H_2_O	35 μl for standard curve, not for testing tissue extract
3 M NaCl	10 μl
rPrPsen (0.3 mg/ml)	34 μl (final 0.1 mg/ml)
1 mM thioflavin T	1 μl
seed	10 μl for standard curve, 45 μl for testing tissue extract
total volume of each RT-QuIC	100 μl

### PK-digestion and immunoblot

The 10% brain homogenates of RML- and ME7-infected mice and purified RML- and ME7-PrP^27-30^ were digested with 100 μg/ml of proteinase-K (Roche, Switzerland) at 37°C for 1 h. The digestions were stopped by heating at 100°C for 10 min with 2 × loading buffer. The proteins were separated in a 15% SDS-PAGE followed by transfer to a PVDF membrane (Millipore, USA). The membrane was blocked with 5% non-fat milk for 1 h at room temperature. The proteins were detected with mouse-PrP specific 4H11 monoclonal antibody (a gift from E. Kremmer, National Research Center for Environment and Health, Munich, Germany). Signals were measured on a Diana III luminescence imaging system (Raytest, Germany).

## Competing interests

The authors declare that they have no competing interests.

## Authors’ contributions

HK conceived the research. SS and GM performed the experiments and analyzed data. HK, SS and AG wrote the manuscript. All authors read and approved the final manuscript.

## Supplementary Material

Additional file 1: Figure S1 Determining the time-span of qRT-QuIC. RT-QuIC reactions were seeded with PrP^27-30^ or PrP^C^ with the indicated amounts. A non-seeded reaction was performed as the control. After 130 h at 37°C, both PrP^C^-seeded and non-seeded reactions showed rising curves, indicating that the spontaneous conversion started at approx. 100 h. Therefore, we chose 90 hours as the maximum time-span of monitoring prion conversion in the qRT-QuIC system. **Figures S2 and S3**: Detecting seeded PrP^27-30^ with RT-QuIC. Purified RML and ME7 PrP^27-30^ and control PrP^C^ with the quantities from 10^-10^ to 10^-16^ g were seeded into reactions independently to perform 90 h of RT-QuIC at 37°C. **Figure S4**: The positive RT-QuIC reactions seeded with PrP^27-30^ (10^-10^ to 10^-15.5^ g for RML and 10^-10^ to 10^-16^ g for ME7) within 90 h are shown. Each scale on the Y-axis represents one effective reaction, the X-axis indicates the required hours corresponding to the reaction. The reactions seeded with 10^-16^ g of RML PrP^27-30^ were negative up to 90 h and thus are not shown in the figure. **Table S1**: Comparing PrP^27-30^ concentrations measured by quantitative RT-QuIC and quantitative immunoblot. **Table S2**: The concentration of PrP^27-30^ in 7 tissues from RML scrapie-infected mice of 7 time-points. **Table S3**: The concentration of PrP^27-30^ in 7 tissues from ME7 scrapie-infected mice of 6 time-points. Click here for file
