# Supplementary material for: Establishing quantitative real-time quaking-induced conversion (qRT-QuIC) for highly sensitive detection and quantification of PrPSc in prion-infected tissues
Source: Acta Neuropathol Commun. 2013 Aug 2;1:44. doi: 10.1186/2051-5960-1-44 (PMC3893511; doi:10.1186/2051-5960-1-44)
Supplement: Additional file 1: Figure S1 — Determining the time-span of qRT-QuIC. RT-QuIC reactions were seeded with PrP27-30 or PrPC with the indicated amounts. A non-seeded reaction was performed as the control. After 130 h at 37°C, both PrPC-seeded and non-seeded reactions showed rising curves, indicating that the spontaneous conversion started at approx. 100 h. Therefore, we chose 90 hours as the maximum time-span of monitoring prion conversion in the qRT-QuIC system. Figures S2 and S3: Detecting seeded PrP27-30 with RT-QuIC. Purified RML and ME7 PrP27-30 and control PrPC with the quantities from 10-10 to 10-16 g were seeded into reactions independently to perform 90 h of RT-QuIC at 37°C. Figure S4: The positive RT-QuIC reactions seeded with PrP27-30 (10-10 to 10-15.5 g for RML and 10-10 to 10-16 g for ME7) within 90 h are shown. Each scale on the Y-axis represents one effective reaction, the X-axis indicates the required hours corresponding to the reaction. The reactions seeded with 10-16 g of RML PrP27-30 were negative up to 90 h and thus are not shown in the figure. Table S1: Comparing PrP27-30 concentrations measured by quantitative RT-QuIC and quantitative immunoblot. Table S2: The concentration of PrP27-30 in 7 tissues from RML scrapie-infected mice of 7 time-points. Table S3: The concentration of PrP27-30 in 7 tissues from ME7 scrapie-infected mice of 6 time-points. [file 2051-5960-1-44-S1.ppt]

## Slide 1
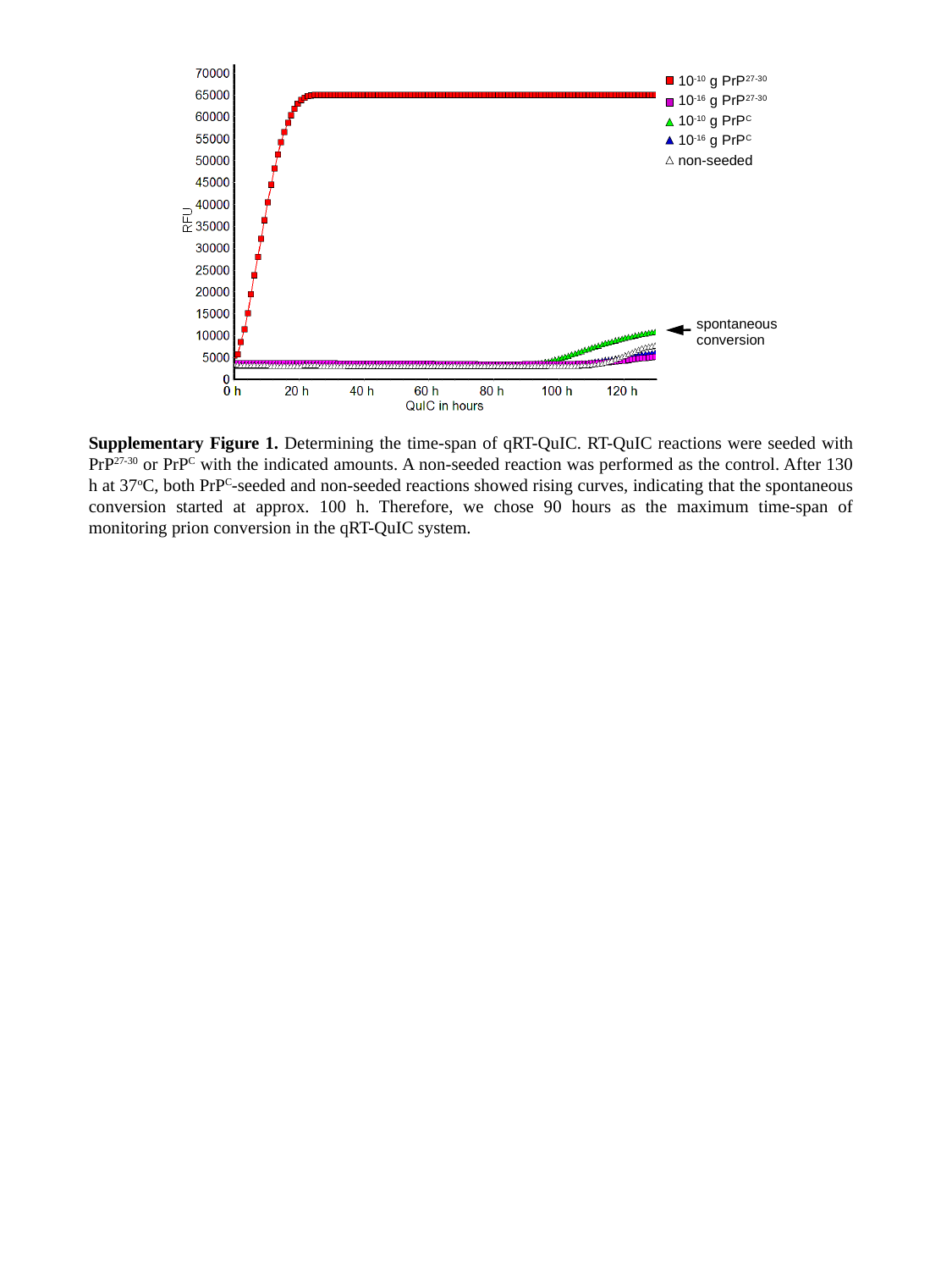

10-10 g PrP27-30
10-16 g PrP27-30
10-10 g PrPC
10-16 g PrPC
non-seeded
spontaneous conversion
Supplementary Figure 1. Determining the time-span of qRT-QuIC. RT-QuIC reactions were seeded with PrP27-30 or PrPC with the indicated amounts. A non-seeded reaction was performed as the control. After 130 h at 37oC, both PrPC-seeded and non-seeded reactions showed rising curves, indicating that the spontaneous conversion started at approx. 100 h. Therefore, we chose 90 hours as the maximum time-span of monitoring prion conversion in the qRT-QuIC system.

## Slide 2
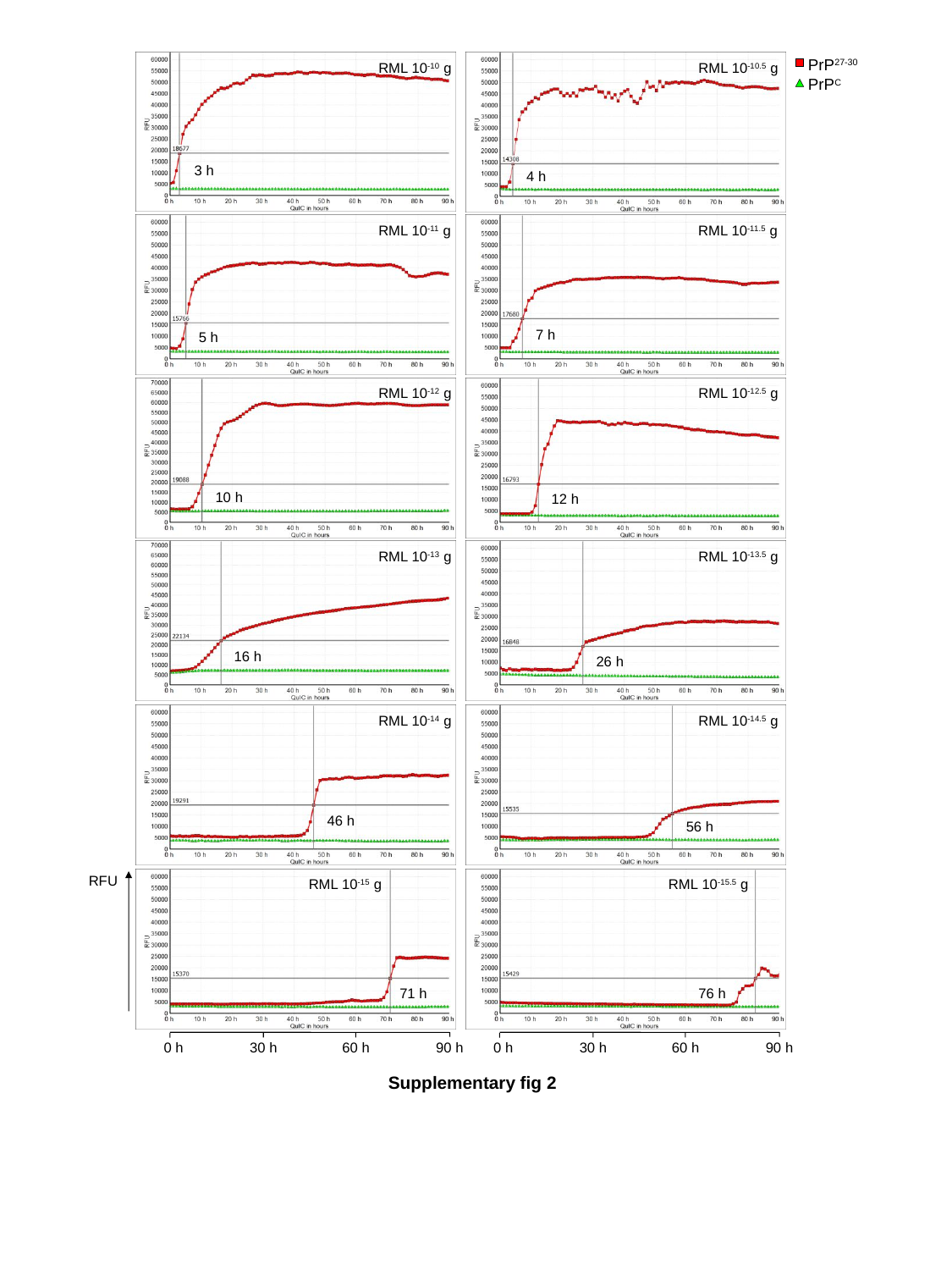

PrP27-30
RML 10-10 g
RML 10-10.5 g
PrPC
3 h
4 h
RML 10-11 g
RML 10-11.5 g
7 h
5 h
RML 10-12 g
RML 10-12.5 g
10 h
12 h
RML 10-13 g
RML 10-13.5 g
16 h
26 h
RML 10-14 g
RML 10-14.5 g
46 h
56 h
RFU
RML 10-15 g
RML 10-15.5 g
71 h
76 h
0 h
30 h
60 h
90 h
0 h
30 h
60 h
90 h
Supplementary fig 2

## Slide 3
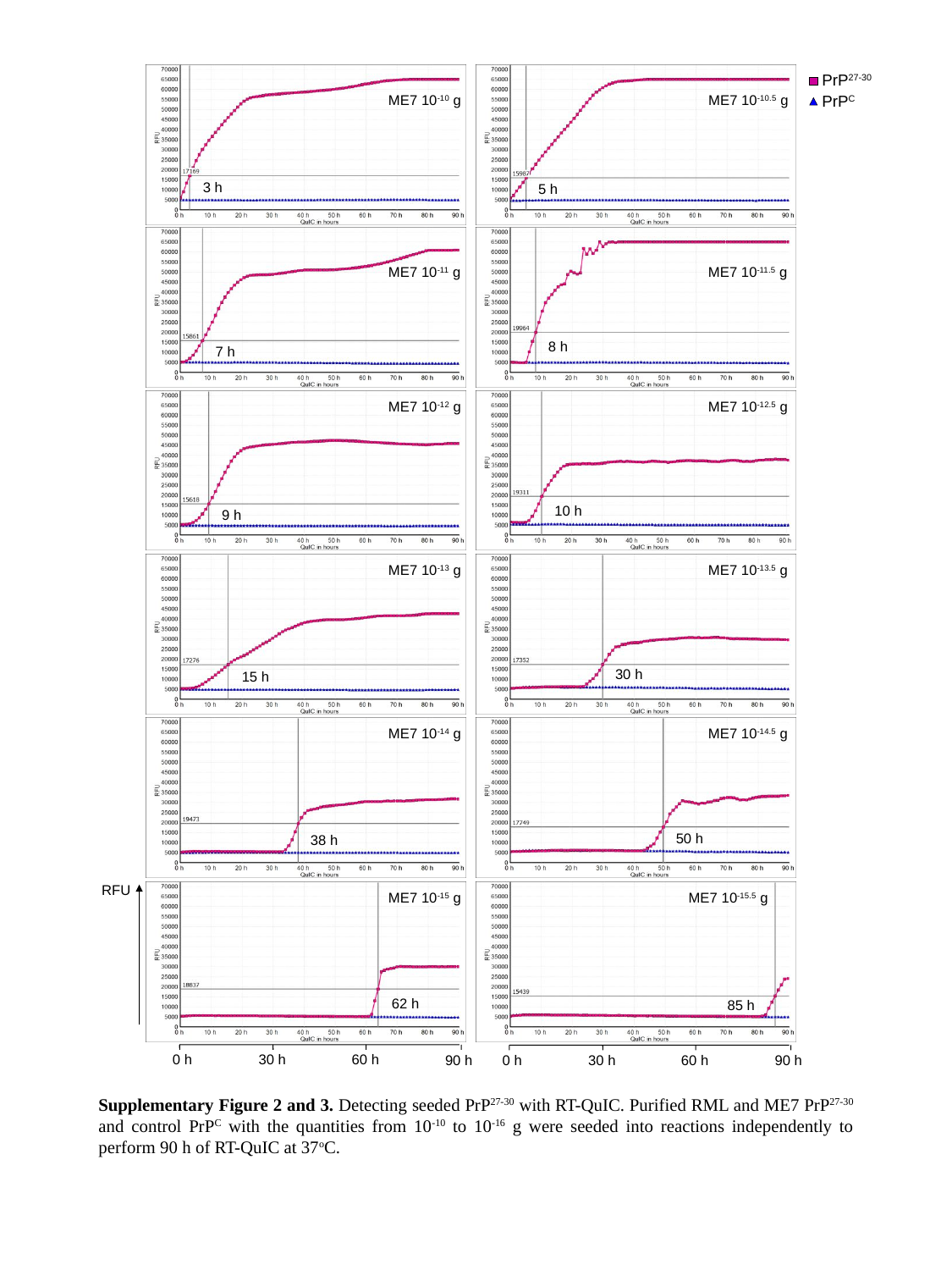

PrP27-30
ME7 10-10 g
ME7 10-10.5 g
PrPC
3 h
5 h
ME7 10-11 g
ME7 10-11.5 g
8 h
7 h
ME7 10-12.5 g
ME7 10-12 g
10 h
9 h
ME7 10-13 g
ME7 10-13.5 g
30 h
15 h
ME7 10-14 g
ME7 10-14.5 g
50 h
38 h
RFU
ME7 10-15 g
ME7 10-15.5 g
62 h
85 h
0 h
30 h
60 h
90 h
0 h
30 h
60 h
90 h
Supplementary Figure 2 and 3. Detecting seeded PrP27-30 with RT-QuIC. Purified RML and ME7 PrP27-30 and control PrPC with the quantities from 10-10 to 10-16 g were seeded into reactions independently to perform 90 h of RT-QuIC at 37oC.

## Slide 4
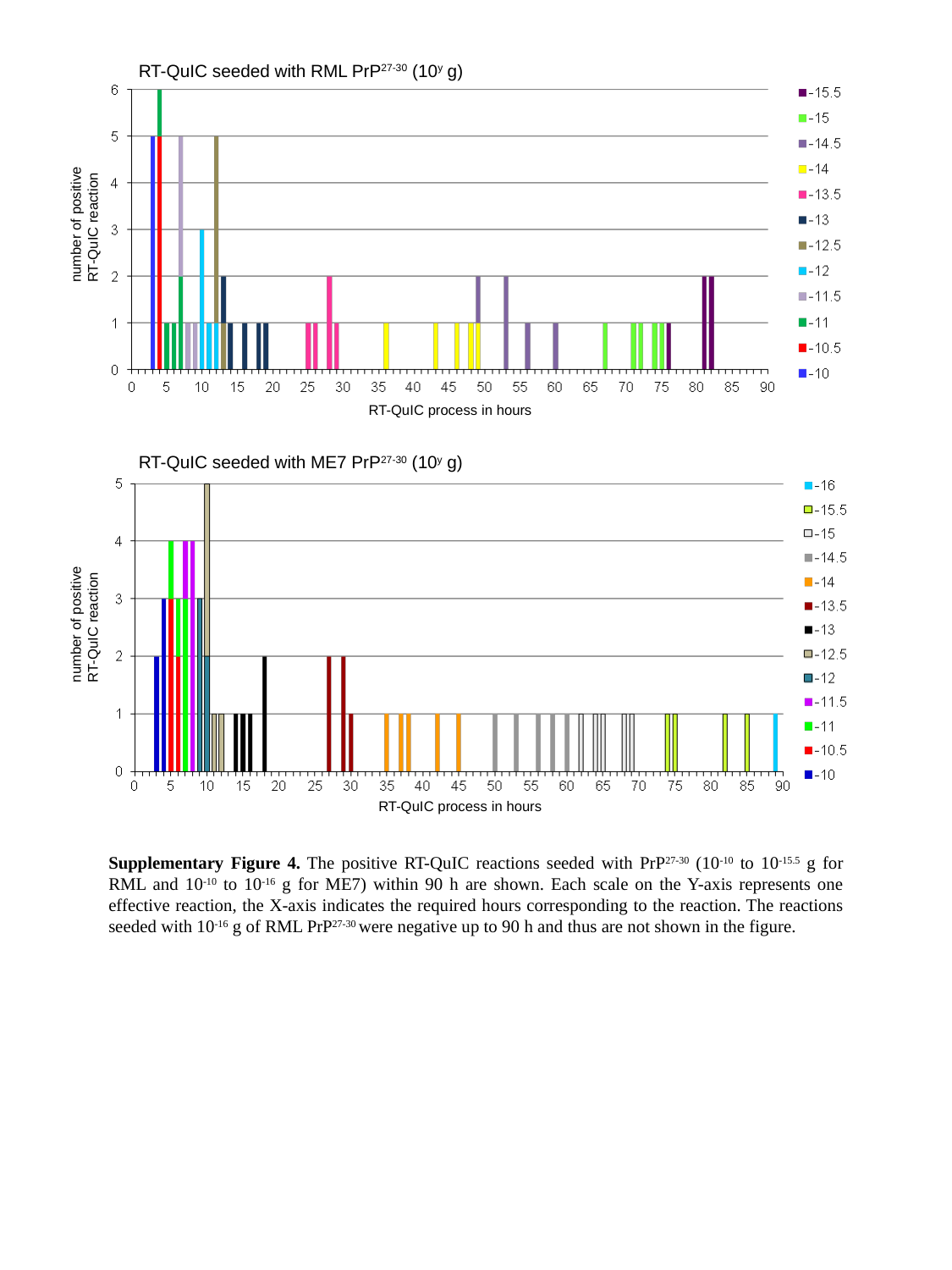

RT-QuIC seeded with RML PrP27-30 (10y g)
number of positive RT-QuIC reaction
RT-QuIC process in hours
RT-QuIC seeded with ME7 PrP27-30 (10y g)
number of positive RT-QuIC reaction
RT-QuIC process in hours
Supplementary Figure 4. The positive RT-QuIC reactions seeded with PrP27-30 (10-10 to 10-15.5 g for RML and 10-10 to 10-16 g for ME7) within 90 h are shown. Each scale on the Y-axis represents one effective reaction, the X-axis indicates the required hours corresponding to the reaction. The reactions seeded with 10-16 g of RML PrP27-30 were negative up to 90 h and thus are not shown in the figure.

## Slide 5
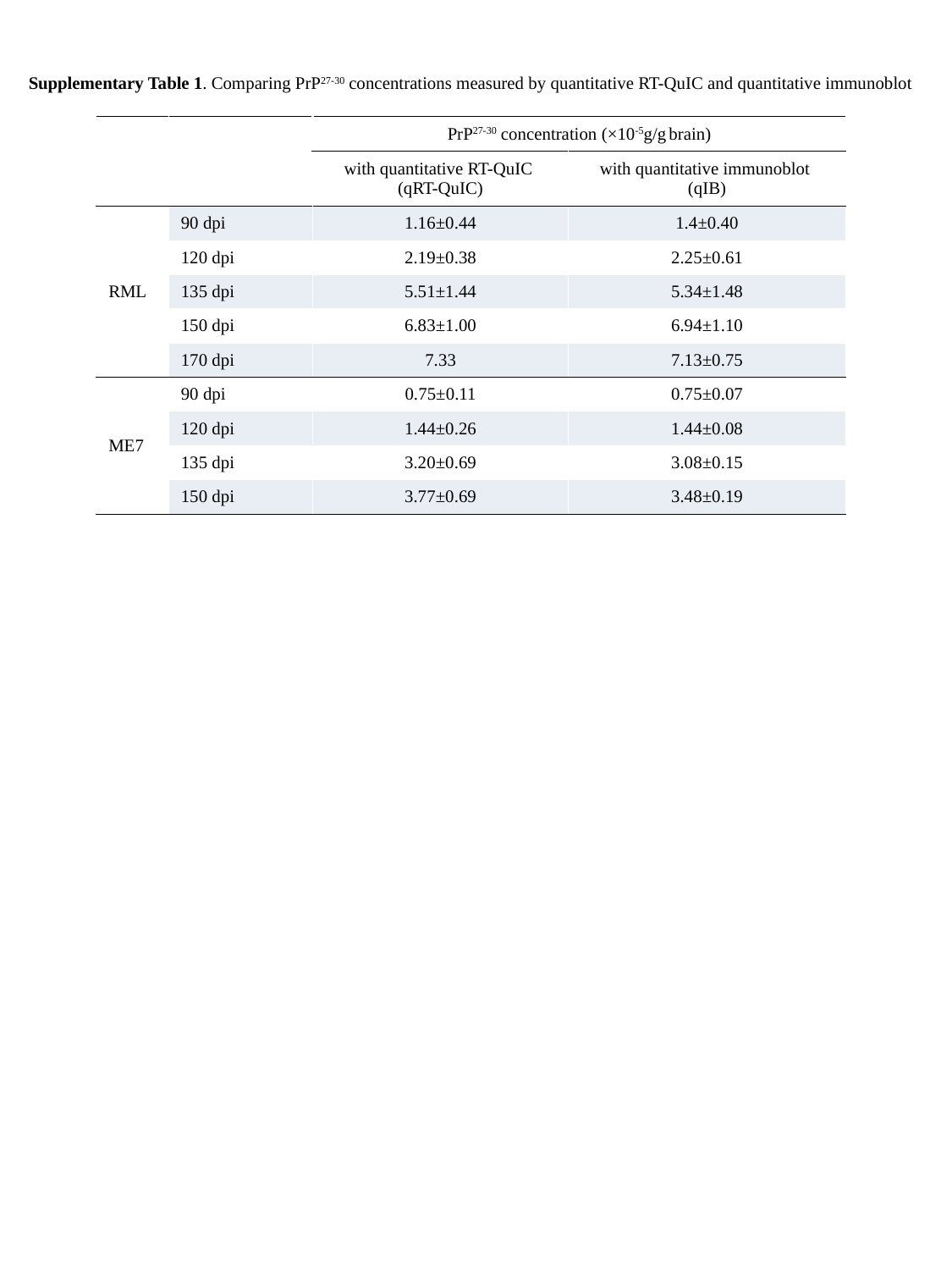

Supplementary Table 1. Comparing PrP27-30 concentrations measured by quantitative RT-QuIC and quantitative immunoblot
| | | PrP27-30 concentration (×10-5g/g brain) | |
| --- | --- | --- | --- |
| | | with quantitative RT-QuIC (qRT-QuIC) | with quantitative immunoblot (qIB) |
| RML | 90 dpi | 1.16±0.44 | 1.4±0.40 |
| | 120 dpi | 2.19±0.38 | 2.25±0.61 |
| | 135 dpi | 5.51±1.44 | 5.34±1.48 |
| | 150 dpi | 6.83±1.00 | 6.94±1.10 |
| | 170 dpi | 7.33 | 7.13±0.75 |
| ME7 | 90 dpi | 0.75±0.11 | 0.75±0.07 |
| | 120 dpi | 1.44±0.26 | 1.44±0.08 |
| | 135 dpi | 3.20±0.69 | 3.08±0.15 |
| | 150 dpi | 3.77±0.69 | 3.48±0.19 |

## Slide 6
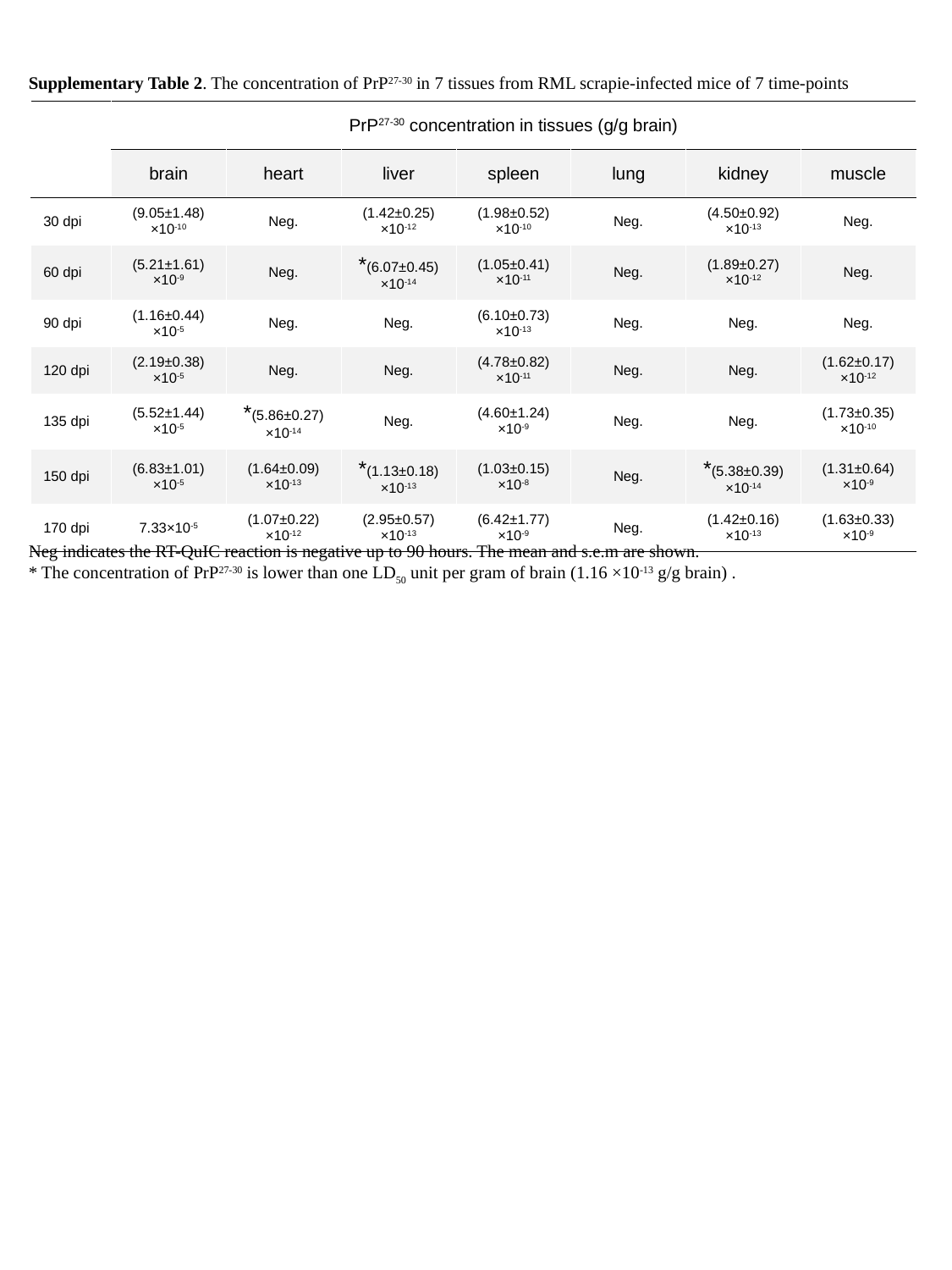

Supplementary Table 2. The concentration of PrP27-30 in 7 tissues from RML scrapie-infected mice of 7 time-points
| | PrP27-30 concentration in tissues (g/g brain) | | | | | | |
| --- | --- | --- | --- | --- | --- | --- | --- |
| | brain | heart | liver | spleen | lung | kidney | muscle |
| 30 dpi | (9.05±1.48) ×10-10 | Neg. | (1.42±0.25) ×10-12 | (1.98±0.52) ×10-10 | Neg. | (4.50±0.92) ×10-13 | Neg. |
| 60 dpi | (5.21±1.61) ×10-9 | Neg. | \*(6.07±0.45) ×10-14 | (1.05±0.41) ×10-11 | Neg. | (1.89±0.27) ×10-12 | Neg. |
| 90 dpi | (1.16±0.44) ×10-5 | Neg. | Neg. | (6.10±0.73) ×10-13 | Neg. | Neg. | Neg. |
| 120 dpi | (2.19±0.38) ×10-5 | Neg. | Neg. | (4.78±0.82) ×10-11 | Neg. | Neg. | (1.62±0.17) ×10-12 |
| 135 dpi | (5.52±1.44) ×10-5 | \*(5.86±0.27) ×10-14 | Neg. | (4.60±1.24) ×10-9 | Neg. | Neg. | (1.73±0.35) ×10-10 |
| 150 dpi | (6.83±1.01) ×10-5 | (1.64±0.09) ×10-13 | \*(1.13±0.18) ×10-13 | (1.03±0.15) ×10-8 | Neg. | \*(5.38±0.39) ×10-14 | (1.31±0.64) ×10-9 |
| 170 dpi | 7.33×10^-5^ | (1.07±0.22) ×10-12 | (2.95±0.57) ×10-13 | (6.42±1.77) ×10-9 | Neg. | (1.42±0.16) ×10-13 | (1.63±0.33) ×10-9 |
Neg indicates the RT-QuIC reaction is negative up to 90 hours. The mean and s.e.m are shown.
* The concentration of PrP27-30 is lower than one LD50 unit per gram of brain (1.16 ×10-13 g/g brain) .

## Slide 7
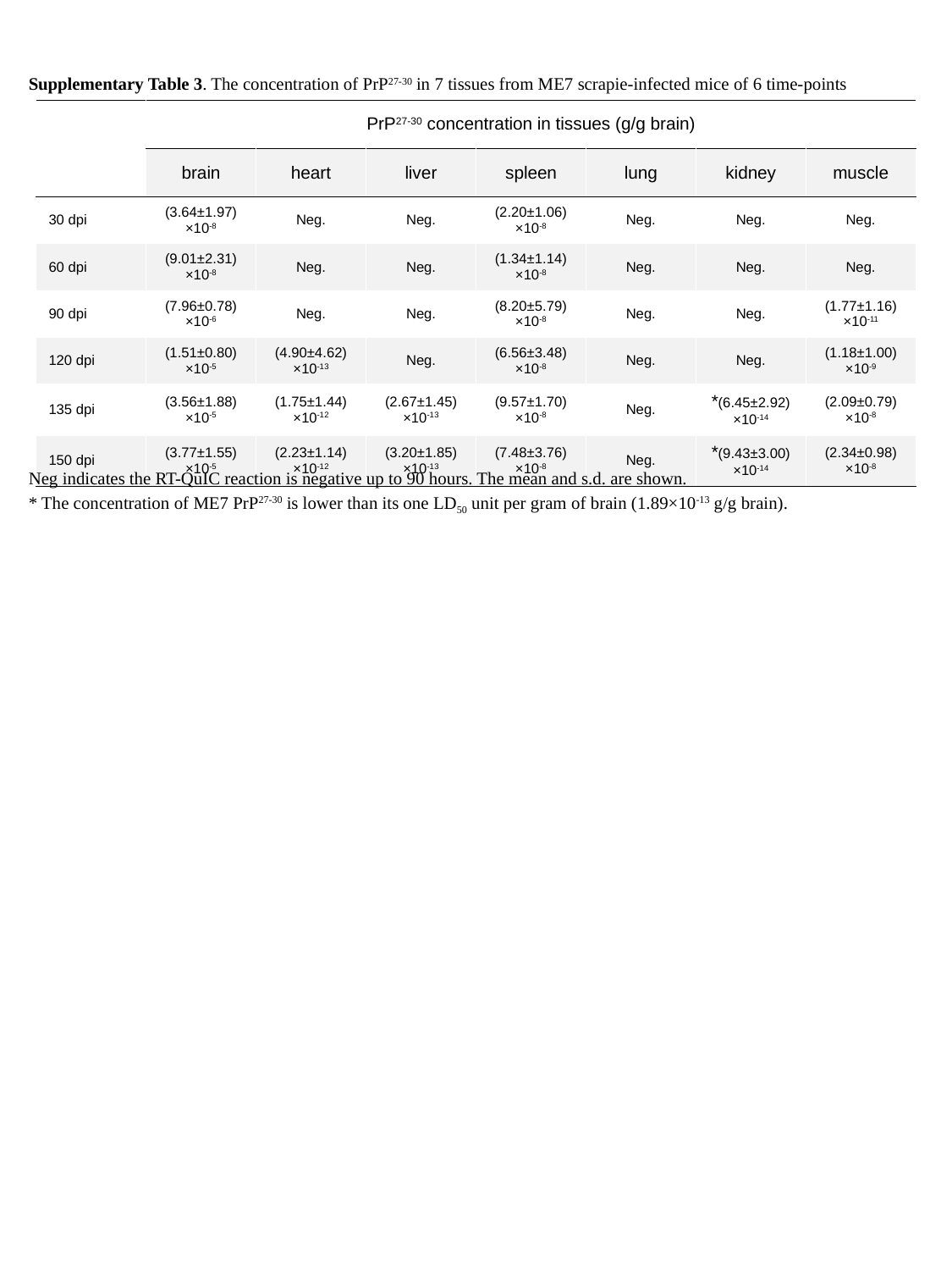

Supplementary Table 3. The concentration of PrP27-30 in 7 tissues from ME7 scrapie-infected mice of 6 time-points
| | PrP27-30 concentration in tissues (g/g brain) | | | | | | |
| --- | --- | --- | --- | --- | --- | --- | --- |
| | brain | heart | liver | spleen | lung | kidney | muscle |
| 30 dpi | (3.64±1.97) ×10-8 | Neg. | Neg. | (2.20±1.06) ×10-8 | Neg. | Neg. | Neg. |
| 60 dpi | (9.01±2.31) ×10-8 | Neg. | Neg. | (1.34±1.14) ×10-8 | Neg. | Neg. | Neg. |
| 90 dpi | (7.96±0.78) ×10-6 | Neg. | Neg. | (8.20±5.79) ×10-8 | Neg. | Neg. | (1.77±1.16) ×10-11 |
| 120 dpi | (1.51±0.80) ×10-5 | (4.90±4.62) ×10-13 | Neg. | (6.56±3.48) ×10-8 | Neg. | Neg. | (1.18±1.00) ×10-9 |
| 135 dpi | (3.56±1.88) ×10-5 | (1.75±1.44) ×10-12 | (2.67±1.45) ×10-13 | (9.57±1.70) ×10-8 | Neg. | \*(6.45±2.92) ×10-14 | (2.09±0.79) ×10-8 |
| 150 dpi | (3.77±1.55) ×10-5 | (2.23±1.14) ×10-12 | (3.20±1.85) ×10-13 | (7.48±3.76) ×10-8 | Neg. | \*(9.43±3.00) ×10-14 | (2.34±0.98) ×10-8 |
Neg indicates the RT-QuIC reaction is negative up to 90 hours. The mean and s.d. are shown.
* The concentration of ME7 PrP27-30 is lower than its one LD50 unit per gram of brain (1.89×10-13 g/g brain).
